# Supplementary material for: Differential Regulation of Hemichannels and Gap Junction Channels by RhoA GTPase and Actin Cytoskeleton: A Comparative Analysis of Cx43 and Cx26
Source: Int J Mol Sci. 2024 Jun 30;25(13):7246. doi: 10.3390/ijms25137246 (PMC11242593; doi:10.3390/ijms25137246)
Supplement: Supplementary file 1 [file ijms-25-07246-s001.zip › ijms-3062450-supplementary.pdf]

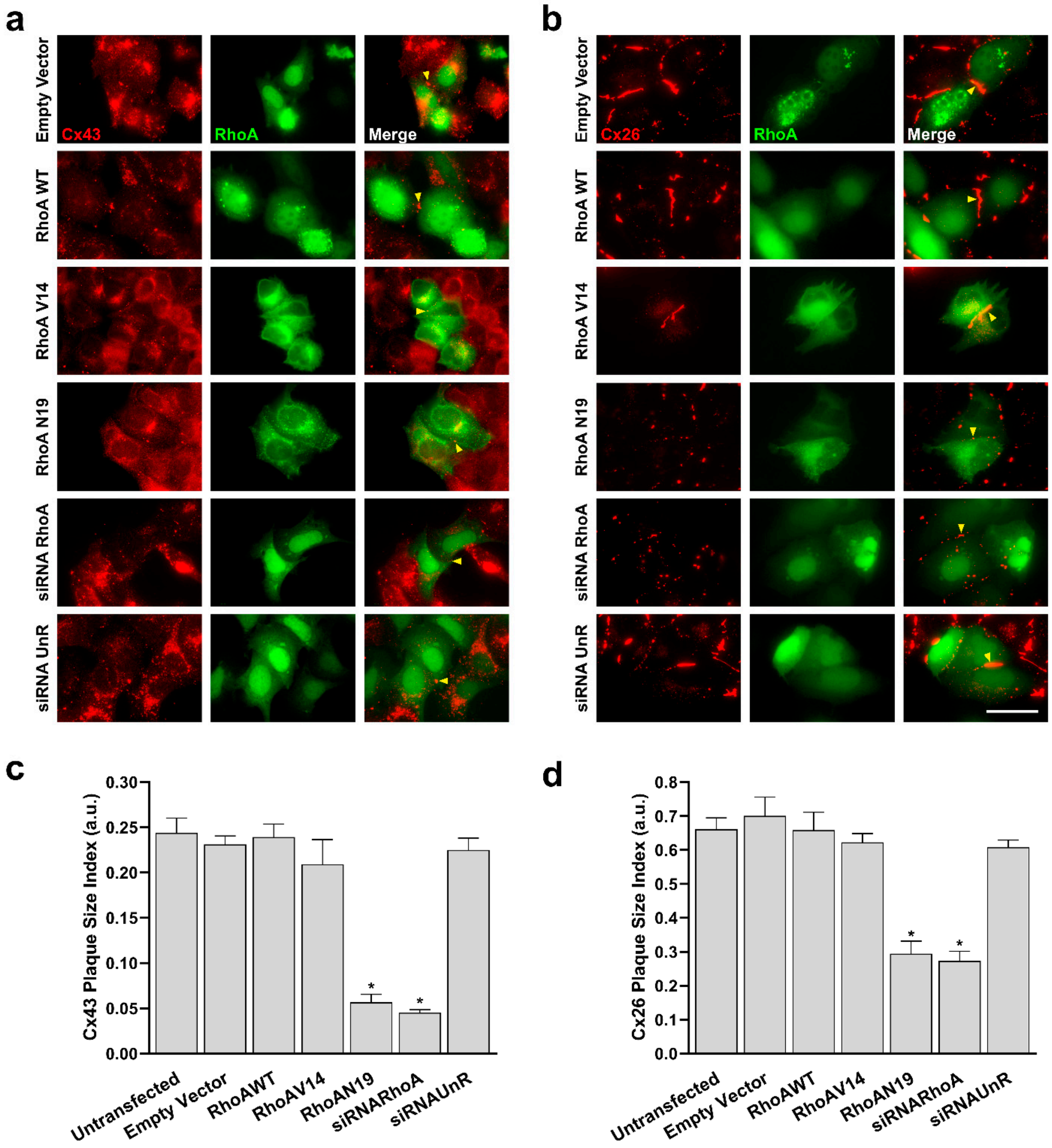

Supplementary Figure S1. Inhibition of RhoA activity or its synthesis significantly reduces the size of Cx43 and Cx26 GJCs plaques. HeLa-Cx43 and HeLa-Cx26 transfected with empty vector or RhoAWT or RhoAV14 or RhoAN19 or siRNARhoA or siRNAUnR constructs (green) were fixed 24 h after transfection and immunolabeling with anti-Cx43 (a) or anti-Cx26 (b) (both shown in red). Quantification of the effect of different constructs on plaque formation at cell-cell borders is shown for Cx43 (c) and Cx26 (d). Data are presented as mean ± SEM (n= 5, 20 cell pairs per condition). All treatments were compared to the control condition (Untransfected) (*p<0.05). Scale bar: 10 µm.

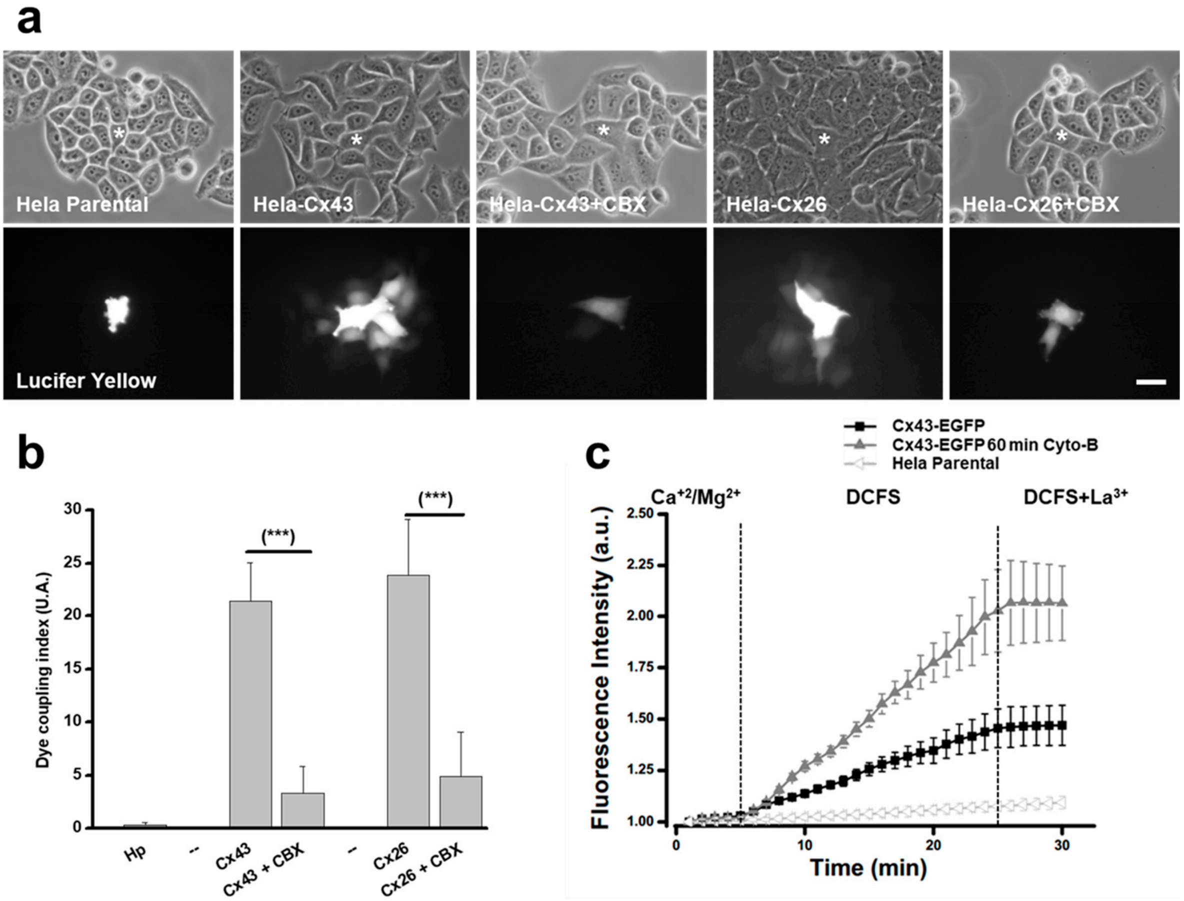

Supplementary Figure S2. Functional HCs and GJCs are not present in HeLa Parental cells. From left to right; phase contrast (upper panels) and Lucifer Yellow (lower panels) microphotographs of HeLa Parental cells, HeLa-Cx43 cells, HeLa-Cx43+CBX, HeLa-Cx26 cells and HeLa-Cx26+CBX (a). Graph shows the dye coupling index in all the evaluated conditions (b). Data are presented as mean ± SEM (n = 3; 6 cells for each condition); *$p<0.001$. Dye uptake induced by DCFS in HeLa cells expressing Cx43EGFP with or without Cyto-B are inhibited with extracellular La^{3+}. Time-lapse of ethidium uptake to address the functional state of HCs. Cells were maintained for 5 min in physiological extracellular divalent cation concentration (Ca^{2+}/Mg^{2+}), followed by a 20 min bath in Ca^{2+}/Mg^{2+} free (DCFS) and finally 100 μM La^{3+} added to block HCs in HeLaCx43EGFP, HeLaCx43EGFP+60 min Cyto-B and HeLa Parental cells (c). Scale bar: 10 μm.

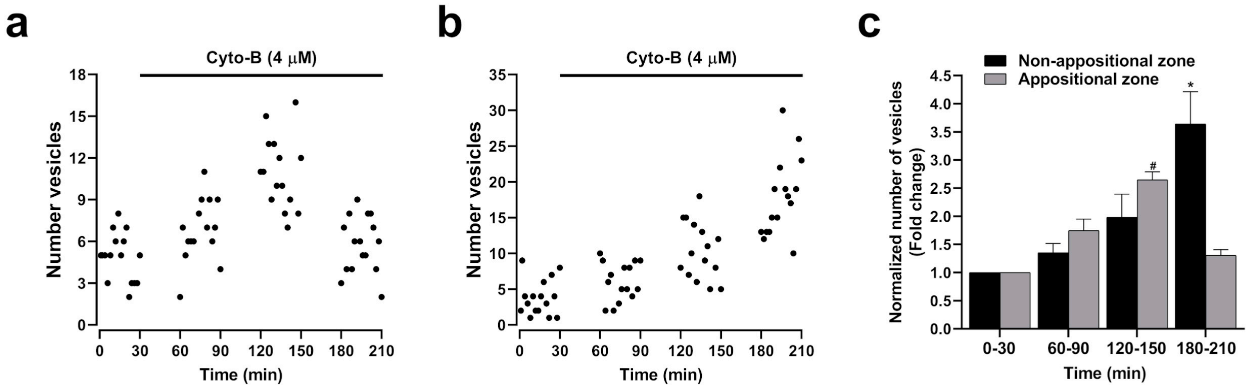

Supplementary Figure S3. Cyto-B increases the number of vesicles in the non-appositional zone in HeLa-Cx26GFP cells. Cells were treated with Cyto-B (4μM) for up to 210 min. Representative graphs show the quantification of the number of vesicles in the appositional zone (a) and the non-appositional zone (b) in HeLa-Cx26GFP cells. The graph shows the fold change in the number of vesicles for the evaluated appositional and non-appositional plasma membranes (c). Non-appositional zone (black bars) time intervals (60-90, 120-150 and 180-210 min) were compared to the control condition (0-30 min). Data are presented as mean ± SEM (n = 3) *$p<0.05$. Appositional zone (gray bars) time intervals (60-90, 120-150 and 180-210 min) were compared to the control condition (0-30 min). Data are presented as mean ± SEM (n = 3) #$p<0.05$.
